# Impact of Tumor-intrinsic Molecular Features on Survival and Acquired Tyrosine Kinase Inhibitor Resistance in ALK-positive NSCLC

**DOI:** 10.1158/2767-9764.CRC-24-0065

**Published:** 2024-03-14

**Authors:** Mari Nakazawa, Guilherme Harada, Paola Ghanem, Adrian Bubie, Lesli A. Kiedrowski, Joseph C. Murray, Kristen A. Marrone, Susan C. Scott, Stefanie Houseknecht, Christina J. Falcon, Patrick Evans, Josephine Feliciano, Christine L. Hann, David S. Ettinger, Kellie N. Smith, Valsamo Anagnostou, Patrick M. Forde, Julie R. Brahmer, Benjamin Levy, Alexander Drilon, Vincent K. Lam

**Affiliations:** 1 Sidney Kimmel Comprehensive Cancer Center, Johns Hopkins University School of Medicine, Baltimore, Maryland.; 2 Memorial Sloan Kettering Cancer Center and Weill Cornell Medical Center, New York, New York.; 3 Guardant Health, Redwood City, California.

## Abstract

While tyrosine kinase inhibitors (TKI) have shown remarkable efficacy in anaplastic lymphoma kinase (*ALK*) fusion-positive advanced non–small cell lung cancer (NSCLC), clinical outcomes vary and acquired resistance remains a significant challenge. We conducted a retrospective study of patients with ALK-positive NSCLC who had clinico-genomic data independently collected from two academic institutions (*n* = 309). This was paired with a large-scale genomic cohort of patients with ALK-positive NSCLC who underwent liquid biopsies (*n* = 1,118). Somatic co-mutations in *TP53* and loss-of-function alterations in *CDKN2A/B* were most commonly identified (24.1% and 22.5%, respectively in the clinical cohort), each of which was independently associated with inferior overall survival (HR: 2.58; 95% confidence interval, CI: 1.62–4.09 and HR: 1.93; 95% CI: 1.17–3.17, respectively). Tumors harboring *EML4-ALK* variant 3 (v3) were not associated with specific co-alterations but were more likely to develop ALK resistance mutations, particularly G1202R and I1171N (OR: 4.11; *P* < 0.001 and OR: 2.94; *P* = 0.026, respectively), and had inferior progression-free survival on first-line TKI (HR: 1.52; 95% CI: 1.03–2.25). Non-v3 tumors were associated with L1196M resistance mutation (OR: 4.63; *P* < 0.001). *EML4-ALK* v3 and somatic co-alterations in *TP53* and *CDKN2A/B* are associated with inferior clinical outcomes. v3 status is also associated with specific patterns of clinically important ALK resistance mutations. These tumor-intrinsic features may inform rational selection and optimization of first-line and consolidative therapy.

**Significance:**

In a large-scale, contemporary cohort of patients with advanced ALK-positive NSCLC, we evaluated molecular characteristics and their impact on acquired resistance mutations and clinical outcomes. Our findings that certain ALK variants and co-mutations are associated with differential survival and specific TKI-relevant resistance patterns highlight potential molecular underpinnings of the heterogenous response to ALK TKIs and nominate biomarkers that may inform patient selection for first-line and consolidative therapies.

## Introduction

The identification of anaplastic lymphoma kinase (*ALK*) fusions as an oncogenic driver in non–small cell lung cancer (NSCLC) has led to the development of potent targeted therapies and in turn revolutionized the treatment of advanced ALK-positive lung cancer (1, 2). *ALK* fusions are found in approximately 4%–6% of lung adenocarcinomas and enriched in younger patients with light or no smoking history (3, 4). In NSCLC, *ALK* is most commonly fused with echinoderm microtubule-associated protein-like 4 (*EML4*), which results in the formation of a chimeric constitutively active protein kinase, capable of activating downstream proliferative pathways including ERK, JAK-STAT, and PI3K-AKT (5). There are at least 12 known variants of *EML4-ALK*, as defined by their fusion breakpoints; most commonly occurring are variant 1 (v1) and variant 3 (v3; ref. 4).

Despite a 5-year overall survival (OS) of over 60% for patients with advanced ALK-positive NSCLC on contemporary tyrosine kinase inhibitors (TKI; ref. 6), development of acquired resistance is inevitable and clinical outcomes remain heterogenous. Acquired resistance to targeted therapy typically occurs after about 3 years and is mediated by both on- and off-target mechanisms (4, 7). The understanding of molecular determinants of ALK TKI clinical outcomes remains sparse. Co-occurring genomic alterations can define molecular subgroups within a specific NSCLC subtype with distinct biology and therapeutic sensitivities (8). Consistent with other oncogenic addicted NSCLC subtypes such as *EGFR*, co-occurring *TP53* mutations appear to be associated with worse prognosis in ALK-positive NSCLC (9–11). However, other co-alterations in important tumor suppressor genes such as *CDKN2A/B* and *SMARCA4* have also been associated with poorer response to TKI in other oncogene-driven tumors (12) but not yet characterized in ALK-positive NSCLC. Furthermore, the pattern of ALK acquired resistance mutations may be influenced by specific ALK variant subtypes (13), though the impact of these variants on clinical outcomes remains uncertain.

Expanding our understanding of these tumor-intrinsic molecular features may facilitate improved patient stratification for the multiple FDA-approved first-line ALK TKIs and potential combination approaches for treatment intensification. In this study, we sought to comprehensively characterize the clinical impact of co-mutations, variants, and other clinical features using a cohort of patients with ALK-positive NSCLC from two major U.S. cancer centers and genomic data from a large-scale international cohort of ALK-positive patients who underwent commercial circulating tumor DNA (ctDNA) testing.

## Materials and Methods

### Clinical Cohort

We retrospectively identified 309 patients with advanced NSCLC at two academic institutions (Johns Hopkins Sidney Kimmel Comprehensive Cancer Center and Memorial Sloan Kettering Cancer Center between January 1, 2010 and December 31, 2022 and March 1, 2005 and August 31, 2021, respectively) harboring somatic ALK fusions from genomic testing of primary tumor specimens or liquid biopsies (Supplementary Fig. S1). *ALK* fusions were detected utilizing tissue or liquid biopsy–based targeted next-generation sequencing (NGS), or through IHC or FISH performed in Clinical Laboratory Improvement Amendments (CLIA)-certified laboratories. Clinical records were reviewed to determine baseline characteristics, treatment history, and clinical outcomes. The primary clinical endpoints of interest included progression-free survival (PFS) on first-line TKI (defined as time to clinical or radiographic disease progression or death due to any cause following initiation of first-line ALK TKI administered for stage IV NSCLC, excluding those who received first-line chemotherapy) and OS (defined as time from stage IV diagnosis to death due to any cause). This retrospective study was approved as exempt by the Institutional Review Boards at Johns Hopkins and Memorial Sloan Kettering (New York, NY). All research was performed in accordance with the U.S. Revised Common Rule.

We assessed the prevalence of co-existing individual clinically relevant somatic alterations, including nonsynonymous missense and nonsense single-nucleotide variants (SNV), insertions, deletions, and frameshift mutations in *TP53*, *PIK3CA*, *APC*, and *CTNNB1*; copy-number loss or loss-of-function mutations in *PTEN*, *CDK4/6*, *CDKN2A/B*, and *CCNE*; and copy-number alterations (CNA) of *MYC* and *MET* (14). These variants were assessed through institutional or commercial CLIA-certified panel NGS platforms**.** Most commonly utilized tissue NGS platforms included the 341-gene Memorial Sloan Kettering-Integrated Mutation Profiling of Actionable Cancer Targets (MSK-IMPACT; ref. 15), 1,000-gene Johns Hopkins Solid Tumor NGS Panel (16), 50-gene Johns Hopkins Limited Solid Tumor NGS Panel (17), 324-gene FoundationOne CDx (F1CDx; ref. 18) and 592-gene Caris Life Sciences Molecular Intelligence Tumor Seek (Caris Life Sciences); all of which use hybridization capture–based tissue NGS, except for the targeted PCR-based Johns Hopkins University NGS Limited platform.

Patients who underwent tissue- or plasma-based NGS that did not cover a mutation of interest were excluded from the analysis of said mutation. The presence of ALK resistance mutations were noted for patients who underwent additional tissue- or plasma-based NGS at the time of disease progression. If multiple instances of NGS testing was pursued at progression (either for the same line of progression or for multiple lines of progression), the union of each unique resistance ALK mutation was attributed to the patient. We applied Cox proportional hazards survival models to assess effect sizes and generate HR based on ALK variant status and associated somatic mutations. Differences between categorical variables were assessed using Fisher exact test.

### Liquid Biopsy Cohort

To further characterize somatic co-mutations and ALK resistance mutations, results from the Guardant Health deidentified clinical database of plasma samples processed between January 1, 2017 and April 1, 2022 were selected for patients with lung cancer with detectable *EML4-ALK*–activating fusion (*n* = 1,350), as defined by the Guardant360 CDx or laboratory developed (LDT) panel tests (collectively G360; ref. 19). The specific timeframe was selected to capture a contemporary cohort of patients likely treated with second generation or later TKIs. G360 specimens were analyzed for clinically relevant somatic co-mutations and ALK resistance mutations as in the clinical cohort. The G360 CDx and LDT assays report results for SNVs, insertion-deletions, and fusions across up to 83 genes at an average coverage depth of 10,000x, including all *ALK* exonic regions (17). Somatic profiling of copy-number loss alterations was not included in all liquid biopsy samples. Samples were filtered to include only the most recent testing timepoint for all patients in the selected population (*n* = 1,118). Fusion breakpoints for each detected mutation were retained for variant subtyping, and fusions were classified into variants based on the exon-exon annotation (20). Relative clonal structure grouping within co-occurring resistance mutations was assessed by recording the maximum and minimum mutant allele frequency (MAF) of all mutations of interest.

### Data Availability

Data were generated by the authors but are not publicly available due to patient confidentiality and protection of private health information. Deidentified data may be provided upon reasonable request from the corresponding author.

## Results

### Baseline Characteristics and EML4-ALK Variant Status

**FIGURE 1:**
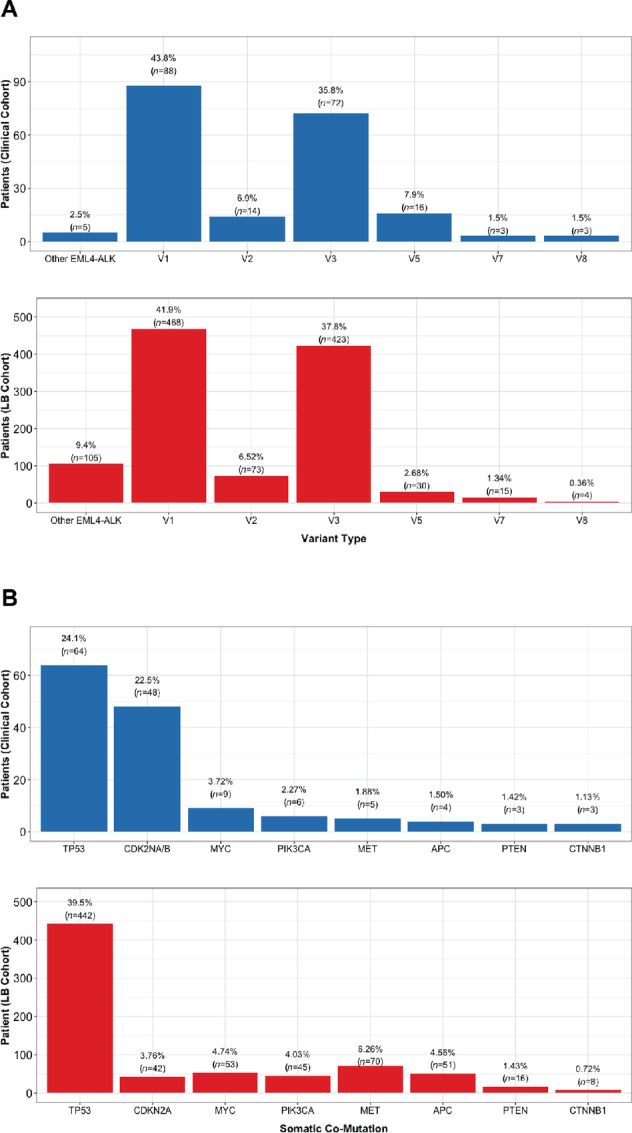
**A,** *EML4-ALK* variant type observed in clinical and liquid biopsy cohorts. **B,** Clinically relevant somatic co-mutations observed in clinical and liquid biopsy (LB) cohorts. Copy-number deletion/loss not reported in the liquid biopsy cohort; loss-of-function mutations in *CDKN2A/B*, *PTEN*, and *CTNNB1* are included.

The clinical cohort included a total of 309 patients (Table 1), 273 of whom underwent tissue NGS at baseline at the discretion of their treating oncologist (Supplementary Fig. S2). In this group, the median age at diagnosis was 53 (range: 16–91). Patients were predominantly female (57.9%), without history of smoking (72.8%), and with stage IV disease (80.7% amongst those with staging information available) at diagnosis. The most common tumor histology was adenocarcinoma (96.8%). Of the 230 patients (74.4%) with known ALK fusion breakpoint, 92.2% harbored an *EML4-ALK* fusion. Of the 201 *EML4-ALK* tumors with known variant, v1 was most common (43.8%) followed by v3 (35.8%; Fig. 1). A total of 7.8% of tumors had *ALK* fusions involving partners other than *EML4*. Most patients received first-line TKI therapy alectinib (57.9%) followed by crizotinib (36.2%). A minority (6.5%) of patients received combination chemotherapy as the first-line treatment for metastatic disease. Of those who had PD-L1 status available, 30.3% had high PD-L1 (>50%) expression. PD-L1 high status appeared to be enriched in v3 patients, though this was not statistically significant (Fisher test OR: 0.53; *P* = 0.10). Overall, baseline clinical characteristics of the v1 and v3 subpopulation were comparable to the overall cohort.

**TABLE 1:** Baseline characteristics of patients with ALK-positive NSCLC in clinical cohort by *EML4-ALK* v1 and v3 subgroups

	*EML4-ALK variant*		
	Variant 1 (*N* = 88)	Variant 3 (*N* = 72)	Clinical cohort (*N* = 309)
Age at diagnosis (years)			
Mean (SD)	50.5 (14.1)	54.0 (14.4)	53.7 (14.3)
Median [Min, Max]	51.0 [24.0, 83.0]	54.0 [16.0, 87.0]	53.0 [16.0, 91.0]
Sex (*n*, %)			
Male	34 (38.6%)	29 (40.3%)	130 (42.1%)
Female	54 (61.4%)	43 (59.7%)	179 (57.9%)
Race (*n*, %)			
White	58 (65.9%)	45 (62.5%)	205 (66.3%)
Black	7 (8.0%)	3 (4.2%)	25 (8.1%)
Asian	16 (18.2%)	13 (18.1%)	48 (15.5%)
Other	7 (8.0%)	11 (15.3%)	31 (10.0%)
Smoking status (*n*, %)			
Never	62 (70.5%)	54 (75.0%)	225 (72.8%)
Former	26 (29.5%)	18 (25.0%)	83 (26.9%)
Not available	0 (0%)	0 (0%)	1 (0.3%)
Histology (*n*, %)			
Adenocarcinoma	86 (97.7%)	67 (93.1%)	299 (96.8%)
Squamous cell carcinoma	1 (1.1%)	0 (0%)	3 (1.0%)
Other	1 (1.1%)	4 (5.6%)	6 (1.9%)
Not available	0 (0%)	1 (1.4%)	1 (0.3%)
Stage at diagnosis (*n*, %)			
I	1 (1.1%)	1 (1.4%)	6 (1.9%)
II	1 (1.1%)	1 (1.4%)	3 (1.0%)
III	8 (9.1%)	5 (6.9%)	18 (5.8%)
IV	37 (42.0%)	39 (54.2%)	113 (36.6%)
Not available	41 (46.6%)	26 (36.1%)	169 (54.7%)
Brain met(s) at stage IV dx (*n*, %)			
No	55 (62.5%)	51 (70.8%)	215 (69.6%)
Yes	33 (37.5%)	21 (29.2%)	94 (30.4%)
PD-L1 ≥50			
No	51 (58.0%)	31 (43.1%)	140 (45.3%)
Yes	14 (15.9%)	16 (22.2%)	61 (19.7%)
Not available	23 (26.1%)	25 (34.7%)	108 (35.0%)
First-line TKI			
Crizotinib	29 (33.0%)	29 (40.3%)	112 (36.2%)
Ceritinib	0 (0%)	0 (0%)	2 (0.6%)
Alectinib	55 (62.5%)	38 (52.8%)	179 (57.9%)
Brigatinib	2 (2.3%)	1 (1.4%)	5 (1.6%)
Lorlatinib	1 (1.1%)	1 (1.4%)	3 (1.0%)
Not available	1 (1.1%)	3 (4.2%)	8 (2.6%)

The liquid biopsy cohort included 1,118 patients with NSCLC harboring an activating *EML4-ALK* fusion, identified from Guardant Health's G360 testing platforms (Supplementary Table S1). These patients were primarily female (56.6%) with a median age of 58 at time of testing. Variants v1 and v3 were most commonly identified (41.9% and 37.8%, respectively). Other variants v2, v5, v7, and v8 collectively accounted for 10.9% of this cohort, while the remaining 9.4% represented other *EML4-ALK* fusions (Fig. 1A).

### Somatic Co-mutations and ALK Resistance Mutations in ALK-positive NSCLC

In addition to characterizing driver *EML4-ALK* fusions, we also assessed the prevalence of clinically relevant co-occurring somatic mutations in our two cohorts. In the clinical cohort, cell cycle and tumor suppressor alterations were most common, driven in large part by *TP53* and *CDKN2A/B* loss co-mutations in 24.1% and 22.5% of patients who underwent NGS, respectively. The most common somatic co-mutation in the liquid biopsy cohort was *TP53*, occurring in 39.5% of specimens (Fig. 1B). There was no correlation between v1 or v3 status and co-occurring somatic mutation in the clinical cohort, while *MYC* amplification was enriched in v1 patients in the liquid biopsy cohort (Fisher test OR 0.47; *P* = 0.02; Supplementary Table S2).

**FIGURE 2:**
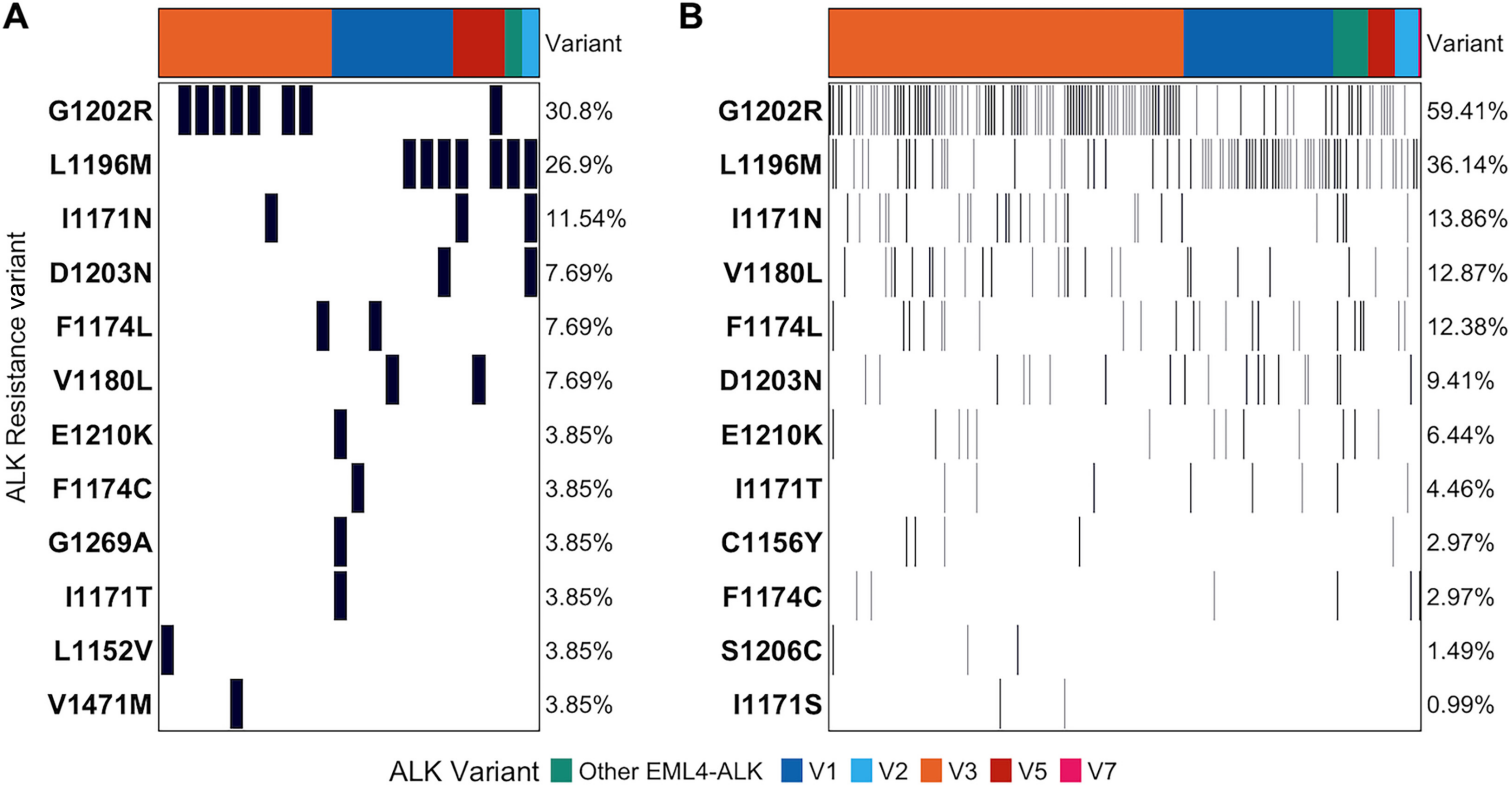
*ALK* resistance mutations observed in clinical (*n* = 26; **A**) and liquid biopsy (*n* = 202; **B**) cohorts, by patient (columns) and grouped by *EML4-ALK* variant type. Filled regions of the oncoprint indicate presence of the *ALK* resistance mutation designated by the row name for a given patient. Resistance mutation percentages indicate share of each among total patients, by cohort. Both G1202R and I1171N are associated with *EML4-ALK* v3 and L1196M is associated with *EML4-ALK* non-v3.

We next sought to explore the relationship between *EML4-ALK* variant type and the emergence of putative ALK resistance mutations in response to TKI treatment. Of the 309 patients in the clinical cohort, 50 patients underwent liquid- or tissue-based NGS at time of progression on TKI therapy, of whom 26 patients (52.0%) were found to have on-target ALK resistance mutations (Fig. 2). Acquired ALK resistance mutations were more likely to occur in v3 patients (Fisher test OR 7.08; *P* = 0.019). The most common resistance mutation were G1202R [seen in 30.8% of patients with resistance ALK mutations and enriched in patients harboring *EML4-ALK* v3 (Fisher test OR 39.2; *P* < 0.001)], and L1196M (identified in 26.9% of patients who had resistance ALK mutations; Fig. 2; Supplementary Table S3).

In the liquid biopsy cohort, we identified 202 patients (18.1%) with an aggregate of 330 on-target ALK resistance mutations. G1202R (59.4%), L1196M (36.1%), and I1171N (13.9%) were the most common *ALK* kinase domain mutations detected (Fig. 2). Resistance mutations were most commonly identified in v3 patients (59.9%), followed by v1 (25.2%). Specifically, *ALK* G1202R and I1171N were both significantly associated with v3 compared with all other variant types (Fisher test OR 4.11; *P* < 0.001 and OR: 2.94; *P* = 0.026, respectively), while *ALK* L1196M was more common among non-v3 patients (Fisher test OR 0.22; *P* < 0.001). We found no other significant associations between v3 and all other variant types for other ALK resistance mutations for which we had sufficient power to test (Supplementary Table S3). We also assessed the association of ALK resistance mutations with co-existing somatic mutations (Supplementary Table S4). We observed that baseline *PIK3CA* co-mutations were associated with development of ALK resistance mutations (Fisher test OR 2.12; *P* = 0.028). Collectively, Wnt/β-catenin/PIK3CA pathway mutations were enriched in ALK-resistance positive specimens (Fisher test OR 2.17; *P* = 0.001), agnostic of variant subtype. No other somatic co-mutations were associated with acquired on-target ALK resistance.

### Multiple Resistance Mutations in ALK-positive NSCLC

In our clinical cohort, of the 26 cases found to have acquired ALK resistance mutations, 3 (11.5%) were found to have multiple ALK resistance mutations. In the liquid biopsy cohort, which included 202 patients with ALK resistance mutations, 80 samples (39.7%) were found to harbor multiple ALK resistance mutations (Supplementary Fig. S3). Notably, D1203N was observed almost exclusively in the context of multiple co-occurring ALK resistance mutations. We found no significant difference in the prevalence of multiple ALK resistance mutations between *EML4-ALK* v1 or v3, the two variants for which we had adequate power for comparison (Supplementary Fig. S3B).

In addition, we assessed whether these multiple ALK mutations in the liquid biopsy cohort were likely to be clonal or nonclonal events (Supplementary Fig. S4). As an estimation, clonal events were defined in this context by a mutation cluster with a maximum MAF range of <5% across resistance mutations. Of the patients with multiple mutations, a majority (*n* = 63, 78.8%) had putative clonal resistance events. However, we did not find any association between resistance mutation structural clonality and variant type (χ^2^ = 0.061; *P* = 0.805), indicating that the emergence of multiresistance clones is likely not related to the *EML4-ALK* isoform differences.

### Impact of Genomic and Molecular Features on Clinical Outcomes

**FIGURE 3:**
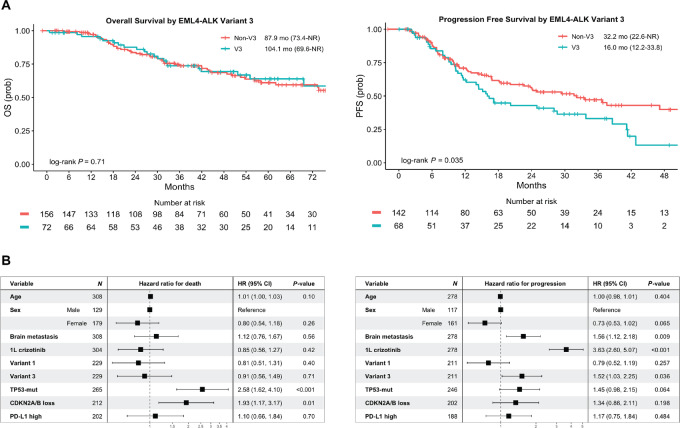
Kaplan–Meier curve for OS and PFS on first-line TKI by EML4-ALK v3 status (**A**). **B,** OS and PFS subgroup analysis by Cox regression. HR, unadjusted hazard ratio; CI, confidence interval.

Finally, we sought to determine the impact of these genomic and molecular features on clinical outcomes. Median OS [84.9 months (95% confidence interval, CI: 73.8–not reached, NR)], PFS on first-line TKI [29.4 months (95% CI: 21.1–37.6)], PFS on first-line crizotinib [12.2 months (95% CI: 9.6–16.2)], and PFS on first-line alectinib or brigatinib [42.8 months (95% CI: 38.6-NR)] in our clinical cohort are consistent with expected ALK-positive NSCLC clinical outcomes. *EML4-ALK* variant status was not associated with differences in OS. In contrast, PFS on first-line TKI was shorter for those who harbored *EML4-ALK* v3 [16.0 months (95% CI: 12.2–33.8) vs. 32.2 months (95% CI: 22.6–NR), unadjusted HR: 1.52; 95% CI: 1.03–2.25] (Fig. 3A). This negative predictive effect remained when restricting the analysis to the more contemporary subgroup of patients that received first-line alectinib and brigatinib [38.6 months (95% CI: 15.7–NR) vs. 51.2 months (95% CI: 37.6–NR), unadjusted HR: 1.78; 95% CI: 0.98–3.24; Supplementary Fig. S5A]. Supplementary Table S5 summarizes the unadjusted and adjusted HR for OS and PFS based on v3 and other select clinical and molecular features utilizing Cox proportional hazards models.

**FIGURE 4:**
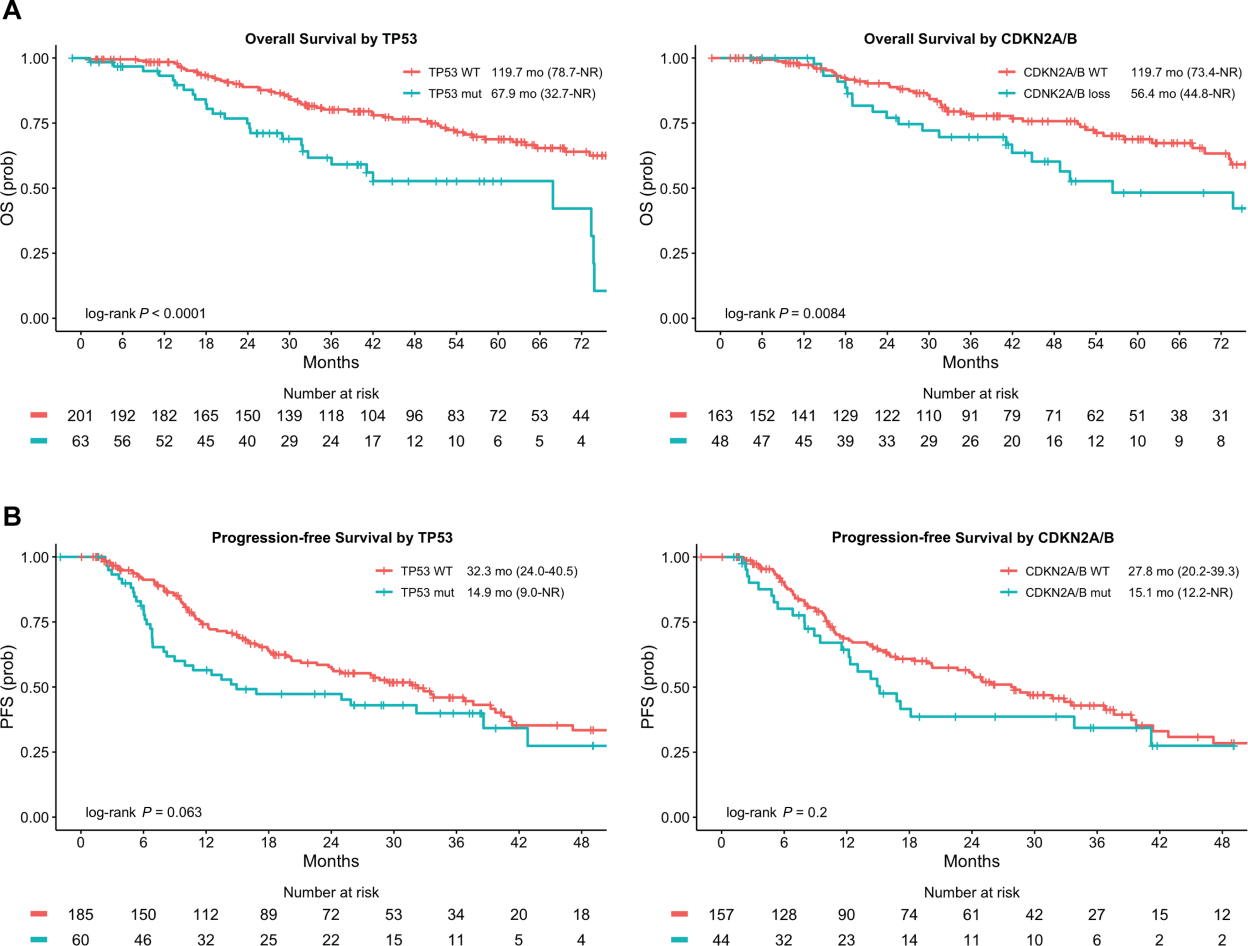
Kaplan–Meier curve for OS (**A**) and PFS (**B**) on first-line TKI by *TP53* mutation and *CDKN2A/B* loss.

Co-existing pathogenic *TP53* mutations or *CDKN2A/B* loss was associated with inferior OS (unadjusted HR: 2.58; 95% CI: 1.62–4.10 and HR: 1.93; 95% CI: 1.17–3.17, respectively; Figs. 3B and 4A). The same trend was observed for PFS on first-line TKI (unadjusted HR: 1.45; 95% CI: 0.98–2.15 and HR: 1.34; 95% CI: 0.86–2.11, respectively for *TP53* and *CDKN2A/B* mutations; Figs. 3B and 4B). Furthermore, in a multivariate model accounting for factors with known PFS significance (brain metastasis and use of first-generation TKI crizotinib), the presence of *TP53* co-mutation remained highly predictive of poorer first-line TKI PFS (adjusted HR: 2.72; 95% CI: 1.48–4.97; Supplementary Table S5B). In the subgroup of patients who received first-line alectinib or brigatinib, numerically worse PFS was observed for those that harbored concurrent *TP53* mutations [42.8 months (95% CI: 25.0–NR) vs. NR (95% CI: 41.2–NR), unadjusted HR: 1.63; 95% CI: 0.89–2.98] but this adverse effect was further attenuated when we evaluated *CDKN2A/B* loss [41.2 months (95% CI: 16.8–NR) vs. 51.2 months (95% CI: 37.6–NR), unadjusted HR: 1.14; 95% CI: 0.54–2.41; Supplementary Table S5C; Supplementary Fig. S5B–S5C].

Finally, while high PD-L1 expression was not associated with OS or PFS outcomes on first-line TKI, the subgroup that received first-line alectinib or brigatinib had numerically inferior PFS [42.8 months (95% CI: 30.7–NR) vs. NR (95% CI: 38.6–NR)] (Supplementary Fig. S6).

## Discussion

In this study, we leveraged the largest cohort to date of patients with advanced ALK-positive NSCLC with genomic profiling data to evaluate molecular characteristics and their impact on acquired resistance mutations and clinical outcomes. We identified *EML4-ALK* v3 as a common fusion variant that is uniquely associated with specific acquired resistance patterns and differences in PFS on first-line TKI. In addition, we identified co-alterations in *TP53* and *CDKN2A/B,* as well as high PD-L1 expression as each associated with inferior clinical outcomes. These findings highlight potential molecular underpinnings to the heterogenous response to first-line ALK TKIs and nominate biomarkers that may inform patient selection for first-line and consolidative therapies.

With clinico-genomic data from a more contemporary cohort of patients primarily treated with second-generation TKIs, we were able to affirm and extend prior observations about the propensity of *EML4-ALK* v3 to develop specific resistance mutations, including strong associations not previously described involving specific resistance mutations I117N and L1196M (13). We found that v3 is not only associated with the highly resistant G1202R acquired mutation but also with I1171N, which is uniquely resistant to alectinib but sensitive to other second-generation TKIs like brigatinib and ceritinib (21). In addition, non-v3 tumors (primarily v1) are associated with L1196M, an acquired gatekeeper mutation that is similarly resistant to alectinib but sensitive to other second-generation TKIs (21, 22). We observed that multiple ALK resistance mutations were present at a prevalence of about 40% at the time of acquired ALK-dependent resistance, affirming a prior single-institution series of post-lorlatinib tissue NGS results (23). These co-occurring resistance mutations appear to primarily be clonal events suggestive of compound resistance mutations that are likely to arise as a result of sequential ALK TKI therapy (24).

Consistent with exploratory analyses from the ALTA-1 L and ALEX randomized phase III studies, we found that *EML4-ALK* v3 was associated with shorter first-line PFS in our clinical cohort (25, 26). Similar to ALTA-1L, there was no difference in OS of v3 patients, suggesting the effectiveness of later lines of therapy, such as lorlatinib, in addressing differentially acquired resistance mutations such as G1202R and I1171N (27). The underlying biology for this *EML4-ALK* v3 difference in TKI sensitivity remains incompletely understood. Compared with non-v3 variants, alternative splicing in v3 appears to result in increased isoform heterogeneity that may be associated with worse clinical reponse to crizotinib (28). Provocatively, cell line inhibition experiments have shown decreased crizotinib sensitivity to the v3a isoform compared with v3b and enrichment of v3a-expressing cells with crizotinib, certinib, or alectinib exposure (28–30). Supported by these prior data, our clinical findings have implications in a setting where the optimal sequencing of ALK TKIs remains an open question. Lorlatinib, a third-generation TKI, has more recently become available as a first-line TKI option for advanced ALK-positive NSCLC, though concerns about toxicity have led to significant reticence in its adoption until more mature survival data emerge (31). Our clinical findings suggest that *EML4-ALK* variant type may be an important predictive biomarker to guide first-line TKI selection or risk stratification in future prospective studies. In this framework, patients with v3 might be prioritized for a more potent third-generation TKI such as lorlatinib, whereas non-v3 patients may still have durable first-line PFS with a less toxic, L1196M-sensitive second-generation TKI (e.g., brigatinib, ceritinib, or ensartinib).

In our clinical cohort, we identified *TP53* mutations and *CDKN2A/B* loss as independent adverse prognostic factors. *TP53* co-mutations occur in up to 40% of patients with advanced ALK-positive NSCLC and is associated with inferior OS and PFS (9–11, 25). *CDKN2A/B* loss is less commonly observed in NSCLC, with inactivating mutations or focal deletions in chromosome 9p occurring in about 12% of advanced NSCLC (32). The significance of somatic *CDKN2A/B* loss in patients with ALK-positive NSCLC is less well described, but has recently been reported to confer worse survival and higher risk of brain metastases (33). The latter finding has been supported by work showing enrichment of *CDKN2A/B* loss in metastatic brain lesions compared with primary tumor specimens in NSCLC (34). Interestingly, the deleterious effect of *CDKN2A/B* loss is not seen with our PFS analysis, especially in those who received second-generation TKIs, perhaps owing to the improved central nervous system protection offered by these agents.

Our liquid biopsy cohort identified *PIK3CA* mutations and, more broadly, PIK3CA/Wnt/β-catenin pathway alterations to be associated with ALK resistance mutations. These mutations have not been well characterized in the landscape of ALK-positive disease, but do not appear to be enriched in progression specimens from patients with *EGFR*-mutated NSCLC (35). The potential relevance of these mutations in mediating ALK TKI resistance and other adverse clinical outcomes warrants further investigation, especially in light of previous work showing cooperative, non-redundant functions in these pathways in promoting *EGFR*-mutated tumor progression (14).

Our group and others have shown that ALK-positive NSCLC has relatively high tumor PD-L1 expression (36, 37). This is thought to be due to the intrinsic upregulation of PD-L1 via *EML4-ALK* effects on downstream *PI3K*-*AKT* and *MEK*-*ERK* signaling (38) and, importantly, not indicative of anti-PD(L)1 immune checkpoint inhibitor sensitivity (39). While high PD-L1 expression has been associated with primary resistance to TKIs in *EGFR*-mutated NSCLC (40), its reported impact on TKI clinical outcomes in ALK-positive NSCLC appears more heterogenous (41, 42). In our current study, we observed that patients with PD-L1 high tumors on first-line alectinib or brigatinib have a numerically shorter PFS. This trend was attenuated when accounting for variant type, *TP53* mutational status, and brain metastasis in our multivariate analysis, suggesting that PD-L1 status is not an independent determinant of ALK TKI outcomes.

A limitation of our observational clinical cohort is the heterogeneity of tissue NGS platforms included, resulting in variable coverage of the somatic alterations of interest. Particularly, reporting of CNAs is not standardized across NGS platforms; as a result, for example, loss of *CDKN2A/B* may be underestimated in both the clinical and liquid biopsy cohorts. In addition, the decision to perform NGS testing at disease progression was made at treating physician's discretion, resulting in only a subset of patients undergoing such testing in our clinical cohort. This may bias the acquired resistance alterations observed in our clinical cohort, potentially with overrepresentation of patients with aggressive or higher burden of disease more readily detected by the liquid biopsy testing most commonly used upon progression (as opposed to tissue NGS). We also acknowledge the limitation in standardized assessment of endpoints such as progression from retrospective data, highlighting the need for prospective studies assessing the contribution of variant status, concurrent somatic mutation status, and PD-L1 expression in outcomes on ALK TKI.

## Conclusions

In this large clinical cohort of patients with ALK-positive NSCLC primarily treated with alectinib, we found that *EML4-ALK* v3 status and somatic co-mutations in *TP53* and *CDKN2A/B* were associated with inferior clinical outcomes and v3 status is associated with specific, TKI-relevant ALK resistance patterns. As ALK-positive NSCLC is often diagnosed solely by FISH or IHC, our study highlights the importance of comprehensive genomic tumor profiling at the time of diagnosis to identify patients at most risk for developing early disease progression and provide an opportunity to optimize first-line and potential consolidative therapy in ALK-positive NSCLC.

## Supplementary Material

Supplemental Table 1 Baseline characteristics of patients with ALK-positive NSCLC in liquid biopsy cohort

Supplemental Table 2 Correlation of variant status (v3 vs non-v3) with somatic co-mutation in liquid biopsy cohort

Supplemental Table 3 Distribution of resistance ALK mutations by EML4-ALK variant subtype in (A) clinical and (B) liquid biopsy cohorts. P-value is derived from Fisher's test of v3 vs non-v3. Significance at the level of p < 0.05 is indicated by the asterisk (*).

Supplemental Table 4 Association of specific co-alterations with presence of ALK resistance mutations and variant type in EML-4-ALK samples (n=1118). Co-alterations of interest were (A) TP53 mutation, (B) PIK3CA mutation, (C) Wnt/B-catenin/PIK3CA pathway mutations [APC, CTNNB1, PIK3CA], (D) MET or MYC amplifications, and (E) cell cycle loss of function alterations [CDK4/6 or CDK2NA/B loss or loss of function mutations]. P-value is derived from Fisher's test. Significance at the level of p < 0.05 is indicated by the asterisk (*). Abbreviations: WT, wild-type.

Supplemental Table 5 Summary of adjusted and unadjusted hazard ratios (HR) with respective confidence intervals as generated by Cox proportional-hazards survival models. Clinically relevant variables and their relative effect sizes on (A) overall survival (OS), (B) progression-free survival (PFS) on first-line tyrosine kinase inhibitor (TKI), and (C) PFS on first-line alectinib or brigatinib. Significance at the level of p < 0.05 is indicated by the asterisk (*).

Supplemental Figure 1 CONSORT diagram depicting inclusion and exclusion criteria for clinical cohort

Supplemental Figure 2 Distribution of tissue NGS platforms utilized in the clinical cohort. Includes 10 patients who underwent testing with more than one tissue NGS platform. Other commercial NGS platform includes other commercially available platforms. Other institutional NGS platform includes all other academic in house NGS platforms.

Supplemental Figure 3 (A) Samples with single versus multiple ALK resistance mutations in liquid biopsy cohort. (B) Distribution of patients with multiple ALK resistance mutations across the ALK resistance cohort by fusion variant type, with percentages above each variant type column indicate the proportion of patients with multiple ALK resistance mutations. The inset chi-squared statistic demonstrates no significant association between multiple resistance mutations and variant type. (C) Distribution of most common ALK resistance co-mutation pairs

Supplemental Figure 4 Clonal and non-clonal ALK resistance mutations across patients with multiple resistance mutations. Each ALK mutation is plotted by sample and colored by putative clonal classification based on the maximum difference between resistance mutation mutant allele frequencies (MAFs). Inset table provides the clonal and non-clonal patient counts for those with v1/v3 variant types. Chi-squared statistic indicates there is no significant association with resistance clonality and fusion type.

Supplemental Figure 5 Kaplan-Meier curve for progression-free survival (PFS) on first-line alectinib or brigatinib by (A) variant type, (B) TP53 mutation, and (C) CDKN2A/B mutation status

Supplemental Figure 6 Kaplan-Meier curve for (A) Overall survival (OS), (B) first-line TKI progression-free survival (PFS) and (C) PFS on first-line alectinib or brigatinib by PD-L1 high (TPS > 50%) status
