# Supplementary material for: Impact of Tumor-intrinsic Molecular Features on Survival and Acquired Tyrosine Kinase Inhibitor Resistance in ALK-positive NSCLC
Source: Cancer Res Commun. 2024 Mar 14;4(3):786–95. doi: 10.1158/2767-9764.CRC-24-0065 (PMC10939006; doi:10.1158/2767-9764.CRC-24-0065)
Supplement: Supplemental Table 1 — Baseline characteristics of patients with ALK-positive NSCLC in liquid biopsy cohort [file crc-24-0065-s01.docx]

**Supplemental Table 1:** Baseline characteristics of patients with ALK-positive NSCLC in liquid biopsy cohort

|  |  |  |
| --- | --- | --- |
| **Characteristic** | | **Total, (%)** |
|  | | 1118 |
| Age (at time of testing) | |  |
|  | Median | 58 |
|  | [Q1,Q3] | [48, 67] |
| Sex | |  |
|  | Male | 485 (43.4%) |
|  | Female | 633 (56.6%) |
| *EML4-ALK* Variant Type |  |  |
|  | V1 | 468 (41.9%) |
|  | V2 | 73 (6.52%) |
|  | V3 | 423 (37.8%) |
|  | V5 | 30 (2.68%) |
|  | V7 | 15 (1.34%) |
|  | V8 | 4 (0.36%) |
|  | Other | 105 (9.4%) |
