# Supplementary material for: Impact of Tumor-intrinsic Molecular Features on Survival and Acquired Tyrosine Kinase Inhibitor Resistance in ALK-positive NSCLC
Source: Cancer Res Commun. 2024 Mar 14;4(3):786–95. doi: 10.1158/2767-9764.CRC-24-0065 (PMC10939006; doi:10.1158/2767-9764.CRC-24-0065)
Supplement: Supplemental Table 2 — Correlation of variant status (v3 vs non-v3) with somatic co-mutation in liquid biopsy cohort [file crc-24-0065-s02.docx]

**Supplemental Table 2:** Correlation of variant status (v3 vs non-v3) with somatic co-mutation in liquid biopsy cohort

|  | **V3** | **Not V3** |  | **Fisher's Test** |  |  |
| --- | --- | --- | --- | --- | --- | --- |
| ***TP53* Mut** | 158 | 284 |  | Odds Ratio: | | 0.863 |
| **No *TP53* Mut** | 265 | 411 |  | pval: | | 0.257 |
|  |  |  |  |  | |  |
|  | **V3** | **Not V3** |  | **Fisher's Test** |  |  |
| ***PTEN* Loss** | 2 | 6 |  | Odds Ratio: | | 0.546 |
| **No *PTEN* Loss** | 421 | 689 |  | pval: | | 0.717 |
|  |  |  |  |  | |  |
|  | **V3** | **Not V3** |  | **Fisher's Test** |  |  |
| ***MYC* Amp** | 12 | 41 |  | Odds Ratio: | | 0.466 |
| **No *MYC* Amp** | 411 | 654 |  | pval: | | 0.02 |
|  |  |  |  |  | |  |
|  | **V3** | **Not V3** |  | **Fisher's Test** |  |  |
| ***PIK3CA* Mut** | 16 | 29 |  | Odds Ratio: | | 0.903 |
| **No *PIK3CA* Mut** | 407 | 666 |  | pval: | | 0.876 |
|  |  |  |  |  | |  |
|  | **V3** | **Not V3** |  | **Fisher's Test** |  |  |
| **Cell Cycle Mut** | 15 | 33 |  | Odds Ratio: | | 0.737 |
| **No Cell Cycle Mut** | 408 | 662 |  | pval: | | 0.365 |
|  |  |  |  |  | |  |
|  | **V3** | **Not V3** |  | **Fisher's Test** |  |  |
| **Wnt/B-cat/PIK3CA Mut** | 37 | 61 |  | Odds Ratio: | | 0.996 |
| **No Wnt/B-cat/PIK3CA Mut** | 386 | 634 |  | pval: | | 1 |
