# Supplementary material for: Impact of Tumor-intrinsic Molecular Features on Survival and Acquired Tyrosine Kinase Inhibitor Resistance in ALK-positive NSCLC
Source: Cancer Res Commun. 2024 Mar 14;4(3):786–95. doi: 10.1158/2767-9764.CRC-24-0065 (PMC10939006; doi:10.1158/2767-9764.CRC-24-0065)
Supplement: Supplemental Table 3 — Distribution of resistance ALK mutations by EML4-ALK variant subtype in (A) clinical and (B) liquid biopsy cohorts. P-value is derived from Fisher's test of v3 vs non-v3. Significance at the level of p < 0.05 is indicated by the asterisk (*). [file crc-24-0065-s03.docx]

**Supplemental Table 3:** Distribution of resistance ALK mutations by *EML4-ALK* variant subtype in **(A)** clinical and **(B)** liquid biopsy cohorts. P-value is derived from Fisher's test of v3 vs non-v3. Significance at the level of p < 0.05 is indicated by the asterisk (*)

**A**

| **Characteristic** | | **Total (%)** | **OR (p-value)** | **v3 (%)** | **v1 (%)** |
| --- | --- | --- | --- | --- | --- |
|  |  | 309 |  | 72 | 88 |
| **ALK Resistance Detected** | |  | 7.08 (p=0.019)* |  |  |
|  | Not Detected | 26 (52%) |  | 2 (16.7%) | 12 (63.2%) |
|  | Detected | 24 (48%) |  | 10 (83.3%) | 7 (36.8%) |
|  | Missing | 259 |  | 60 | 69 |
|  |  |  |  |  |  |
| **ALK Resistance Mutation** | | 50 |  | 12 | 19 |
|  | G1202R | 8 (16%) | 39.2 (p=0.0002)* | 7 (58.3%) | 0 (0%) |
|  | L1196M | 6 (12%) | 0 (p=0.16) | 0 (0%) | 3 (15.6%) |
|  | I1171N | 3 (6%) | 1.23 (p=1.00) | 1 (8.3%) | 0 (0%) |
|  | V1180L | 3 (6%) | 0 (p=0.58) | 0 (0%) | 1 (5.3%) |
|  | F1174L | 2 (4%) | 2.55 (p=1.00) | 1 (8.3%) | 1 (5.3%) |
|  | D1203N | 2 (4%) | 0 (p=0.58) | 0 (0%) | 1 (5.3%) |

**B**

| **Characteristic** | | **Total (%)** | **OR (p-value)** | **v3 (%)** | **v1 (%)** |
| --- | --- | --- | --- | --- | --- |
|  | | 1118 |  | 423 | 468 |
| **ALK Resistance Detected** | |  | 3.037 (p<0.0001)* |  |  |
|  | Not Detected | 916 (81.9%) |  | 302 (71.4%) | 417 (89.1%) |
|  | Detected | 202 (18.1%) |  | 121 (28.6%) | 51 (10.9%) |
|  | |  |  |  |  |
| **ALK Resistance Mutation** | | 330 |  | 207 | 71 |
|  | G1202R | 120 (36.4%) | 4.11 (p<0.0001)* | 98 (47.3%) | 9 (12.7%) |
|  | L1196M | 73 (22.1%) | 0.216 (p<0.0001)* | 25 (12.1%) | 34 (47.9%) |
|  | I1171N | 28 (8.48%) | 2.94 (p=0.026)* | 23 (11.1%) | 1 (1.41%) |
|  | V1180L | 26 (7.88%) | 1.67 (p=0.296) | 19 (9.18%) | 4 (5.63%) |
|  | F1174L | 25 (7.58%) | 0.522 (p=0.133) | 12 (5.80%) | 7 (9.86%) |
|  | D1203N | 19 (5.76%) | 0.410 (p=0.084) | 8 (3.86%) | 8 (11.3%) |
|  | E1210K | 13 (3.93%) |  | 6 (2.90%) | 4 (5.63%) |
|  | I1171T | 9 (2.73%) |  | 4 (1.93%) | 3 (4.22%) |
|  | C1156Y | 6 (1.82%) |  | 5 (2.42%) | 0 (0%) |
|  | F1174C | 6 (1.82%) |  | 2 (0.97%) | 1 (1.41%) |
|  | S1206C | 3 (1.01%) |  | 3 (1.45%) | 0 (0%) |
|  | I1171S | 2 (0.61%) |  | 2 (0.97%) | 0 (0%) |
