# Supplementary material for: Impact of Tumor-intrinsic Molecular Features on Survival and Acquired Tyrosine Kinase Inhibitor Resistance in ALK-positive NSCLC
Source: Cancer Res Commun. 2024 Mar 14;4(3):786–95. doi: 10.1158/2767-9764.CRC-24-0065 (PMC10939006; doi:10.1158/2767-9764.CRC-24-0065)
Supplement: Supplemental Table 4 — Association of specific co-alterations with presence of ALK resistance mutations and variant type in EML-4-ALK samples (n=1118). Co-alterations of interest were (A) TP53 mutation, (B) PIK3CA mutation, (C) Wnt/B-catenin/PIK3CA pathway mutations [APC, CTNNB1, PIK3CA], (D) MET or MYC amplifications, and (E) cell cycle loss of function alterations [CDK4/6 or CDK2NA/B loss or loss of function mutations]. P-value is derived from Fisher's test. Significance at the level of p < 0.05 is indicated by the asterisk (*). Abbreviations: WT, wild-type. [file crc-24-0065-s04.docx]

| **A** |  |  |  | **Patient Total (%)** | | **OR (p-value)** |
| --- | --- | --- | --- | --- | --- | --- |
| ***TP53* Mutations** | |  |  |  |  |  |
|  | Mut +, ALK Resistance + | | | 79 (7.0%) | | 0.978 (p=0.937) |
|  | Mut +, ALK Resistance - | | | 363 (32.5%) | |  |
|  | WT, ALK Resistance + | | | 123 (11.0%) | |  |
|  | WT, ALK Resistance - | | | 553 (49.5%) | |  |
| *TP53* WT | | | | 676 (60.5%) | |  |
| *TP53* Mutated | | | | 442 (39.5%) | |  |
|  | V1 | | | 193 | | p=0.593 |
|  | V2 | | | 29 | |  |
|  | V3 | | | 158 | |  |
|  | V5 | | | 14 | |  |
|  | V7 | | | 5 | |  |
|  | V8 | | | 0 | |  |
|  | Other | | | 43 | |  |

**Supplemental Table 4:** Association of specific co-alterations with presence of ALK resistance mutations and variant type in *EML-4-ALK* samples (n=1118). Co-alterations of interest were **(A)** *TP53* mutation, **(B)** *PIK3CA* mutation, **(C)** Wnt/B-catenin/PIK3CA pathway mutations [*APC*, *CTNNB1*, *PIK3CA*], **(D)** *MET* or *MYC* amplifications, and **(E)** cell cycle loss of function alterations [*CDK4/6* or *CDK2NA/B* loss or loss of function mutations]. P-value is derived from Fisher's test. Significance at the level of p < 0.05 is indicated by the asterisk (*). Abbreviations: WT, wild-type.

| **C** |  |  |  | **Patient Total (%)** | | **OR (p-value)** |
| --- | --- | --- | --- | --- | --- | --- |
| **Wnt/B-catenin/PIK3CA Pathway (*APC*, *CTNNB1*, *PIK3CA*) Mutations** | | | |  |  |  |
|  | Mut +, ALK Resistance + | | | 30 (2.68%) | | 2.17 (p=0.001*) |
|  | Mut +, ALK Resistance - | | | 68 (6.08%) | |  |
|  | WT, ALK Resistance + | | | 172 (14.75%) | |  |
|  | WT, ALK Resistance - | | | 848 (75.8%) | |  |
| *APC, CTNNB1, PIK3CA* WT | | |  | 1020 (91.23%) | |  |
| *APC, CTNNB1, PIK3CA* Mutated | | |  | 98 (8.77%) | |  |
|  | V1 |  |  | 45 | | p=0.907 |
|  | V2 |  |  | 5 | |  |
|  | V3 |  |  | 37 | |  |
|  | V5 |  |  | 2 | |  |
|  | V7 |  |  | 2 | |  |
|  | V8 |  |  | 0 | |  |
|  | Other |  |  | 7 | |  |

| **B** |  |  |  | **Patient Total (%)** | | **OR (p-value)** |
| --- | --- | --- | --- | --- | --- | --- |
| ***PIK3CA* Mutations** | |  |  |  |  |  |
|  | Mut +, ALK Resistance + | | | 14 (1.25%) | | 2.12 (p=0.028)* |
|  | Mut +, ALK Resistance - | | | 31 (2.77%) | |  |
|  | WT, ALK Resistance + | | | 188 (16.8%) | |  |
|  | WT, ALK Resistance - | | | 885 (79.2%) | |  |
| *PIK3CA* WT | | |  | 1073(95.9%) | |  |
| *PIK3CA* Mutated | | |  | 45 (4.02%) | |  |
|  | V1 |  |  | 21 | | p=0.754 |
|  | V2 |  |  | 1 | |  |
|  | V3 |  |  | 16 | |  |
|  | V5 |  |  | 2 | |  |
|  | V7 |  |  | 0 | |  |
|  | V8 |  |  | 0 | |  |
|  | Other |  |  | 5 | |  |

| **D** |  |  |  | **Patient Total (%)** | | **OR (p-value)** |
| --- | --- | --- | --- | --- | --- | --- |
| ***MET*/*MYC* Amplifications** | | | |  |  |  |
|  | Amp, ALK Resistance + | | | 16 (1.43%) | | 0.809 (p=0.506) |
|  | Amp, ALK Resistance - | | | 88 (7.87%) | |  |
|  | WT, ALK Resistance + | | | 186 (16.6%) | |  |
|  | WT, ALK Resistance - | | | 828 (74.1%) | |  |
| *MET/MYC* WT | | |  | 1014 (90.7%) | |  |
| *MET/MYC* Amp | | |  | 104 (9.3%) | |  |
|  | V1 |  |  | 49 | | p=0.048* |
|  | V2 |  |  | 11 | |  |
|  | V3 |  |  | 29 | |  |
|  | V5 |  |  | 0 | |  |
|  | V7 |  |  | 2 | |  |
|  | V8 |  |  | 0 | |  |
|  | Other |  |  | 13 | |  |

| **E** |  |  |  | **Patient Total (%)** | | **OR (p-value)** |
| --- | --- | --- | --- | --- | --- | --- |
| **Cell-Cycle Pathway (*CDK4/6, CDK2NA/B*) Loss** | | | |  |  |  |
|  | Loss +, ALK Resistance + | | | 12 (1.07%) | | 1.54 (p=0.247) |
|  | Loss +, ALK Resistance - | | | 36 (3.22%) | |  |
|  | WT, ALK Resistance + | | | 190 (16.9%) | |  |
|  | WT, ALK Resistance - | | | 880 (78.7%) | |  |
| *CDK4/6, CDK2NA/B* WT | | |  | 1070 (95.7%) | |  |
| *CDK4/6, CDK2NA/B* Loss | | |  | 48 (4.3%) | |  |
|  | V1 |  |  | 17 | | p=0.071 |
|  | V2 |  |  | 7 | |  |
|  | V3 |  |  | 15 | |  |
|  | V5 |  |  | 4 | |  |
|  | V7 |  |  | 0 | |  |
|  | V8 |  |  | 0 | |  |
|  | Other |  |  | 5 | |  |
