# Supplementary material for: Impact of Tumor-intrinsic Molecular Features on Survival and Acquired Tyrosine Kinase Inhibitor Resistance in ALK-positive NSCLC
Source: Cancer Res Commun. 2024 Mar 14;4(3):786–95. doi: 10.1158/2767-9764.CRC-24-0065 (PMC10939006; doi:10.1158/2767-9764.CRC-24-0065)
Supplement: Supplemental Table 5 — Summary of adjusted and unadjusted hazard ratios (HR) with respective confidence intervals as generated by Cox proportional-hazards survival models. Clinically relevant variables and their relative effect sizes on (A) overall survival (OS), (B) progression-free survival (PFS) on first-line tyrosine kinase inhibitor (TKI), and (C) PFS on first-line alectinib or brigatinib. Significance at the level of p < 0.05 is indicated by the asterisk (*). [file crc-24-0065-s05.docx]

**Supplemental Table 5:** Summary of adjusted and unadjusted hazard ratios (HR) with respective confidence intervals as generated by Cox proportional-hazards survival models. Clinically relevant variables and their relative effect sizes on **(A)** overall survival (OS), **(B)** progression-free survival (PFS) on first-line tyrosine kinase inhibitor (TKI), and **(C)** PFS on first-line alectinib or brigatinib. Significance at the level of p < 0.05 is indicated by the asterisk (*).

**A**

|  | *Unadjusted* | | | *Adjusted* | | |
| --- | --- | --- | --- | --- | --- | --- |
| **Feature** | **HR** | **95% CI** | **p-value** | **HR** | **95% CI** | **p-value** |
| Age at Dx | 1.01 | 1.00-1.03 | 0.10 |  |  |  |
| Sex | 0.8 | 0.54-1.18 | 0.26 |  |  |  |
| Variant 1 | 0.81 | 0.51-1.31 | 0.40 |  |  |  |
| Variant 3 | 0.91 | 0.56-1.49 | 0.71 |  |  |  |
| Brain mets | 1.12 | 0.76-1.67 | 0.56 |  |  |  |
| PD-L1 high | 1.1 | 0.66-1.84 | 0.70 |  |  |  |
| 1L crizotinib use | 0.85 | 0.56-1.27 | 0.42 | 1.03 | 0.64-1.69 | 0.90 |
| *TP53* mut | 2.58 | 1.62-4.09 | <0.001* | 2.40 | 1.41-4.09 | 0.001* |
| *CDKN2A/B* loss | 1.93 | 1.17-3.17 | 0.01* | 1.71 | 1.02-2.84 | 0.04* |

**B**

|  | *Unadjusted* | | | *Adjusted* | | |
| --- | --- | --- | --- | --- | --- | --- |
| **Feature** | **HR** | **95% CI** | **p-value** | **HR** | **95% CI** | **p-value** |
| Age at Dx | 1 | 0.98-1.01 | 0.40 |  |  |  |
| Sex | 0.73 | 0.53-1.02 | 0.06 |  |  |  |
| Variant 1 | 0.79 | 0.52-1.19 | 0.26 |  |  |  |
| Variant 3 | 1.52 | 1.03-2.25 | 0.04* | 1.63 | 0.98-2.73 | 0.06 |
| Brain mets | 1.56 | 1.12-2.18 | 0.009* | 1.15 | 0.68-1.92 | 0.60 |
| PD-L1 high | 1.17 | 0.75-1.84 | 0.48 | 1.54 | 0.89-2.66 | 0.12 |
| 1L crizotinib use | 3.63 | 2.60-5.07 | <0.001* | 7.20 | 4.03-12.89 | <0.001* |
| *TP53* mut | 1.45 | 0.98-2.15 | 0.06 | 2.72 | 1.48-4.97 | 0.001* |
| *CDKN2A/B* loss | 1.34 | 0.86-2.11 | 0.20 | 1.39 | 0.75-2.60 | 0.30 |

**C**

|  | *Unadjusted* | | | *Adjusted* | | |
| --- | --- | --- | --- | --- | --- | --- |
| **Feature** | **HR** | **95% CI** | **p-value** | **HR** | **95% CI** | **p-value** |
| Age at Dx | 1 | 0.98-1.02 | 0.80 |  |  |  |
| Sex | 0.88 | 0.52-1.52 | 0.66 |  |  |  |
| Variant 1 | 0.57 | 0.29-1.13 | 0.11 |  |  |  |
| Variant 3 | 1.78 | 0.98-3.24 | 0.06 | 2.38 | 1.18-4.78 | 0.02* |
| Brain mets | 1.61 | 0.94-2.74 | 0.08 | 1.47 | 0.73-2.94 | 0.28 |
| PD-L1 high | 1.61 | 0.86-3.00 | 0.13 | 1.40 | 0.70-2.84 | 0.34 |
| *TP53* mut | 1.63 | 0.89-2.98 | 0.11 | 1.85 | 0.92-3.71 | 0.08 |
| *CDKN2A/B* loss | 1.14 | 0.54-2.41 | 0.74 |  |  |  |
