# Supplementary material for: Impact of Tumor-intrinsic Molecular Features on Survival and Acquired Tyrosine Kinase Inhibitor Resistance in ALK-positive NSCLC
Source: Cancer Res Commun. 2024 Mar 14;4(3):786–95. doi: 10.1158/2767-9764.CRC-24-0065 (PMC10939006; doi:10.1158/2767-9764.CRC-24-0065)
Supplement: Supplemental Figure 1 — CONSORT diagram depicting inclusion and exclusion criteria for clinical cohort [file crc-24-0065-s06.docx]

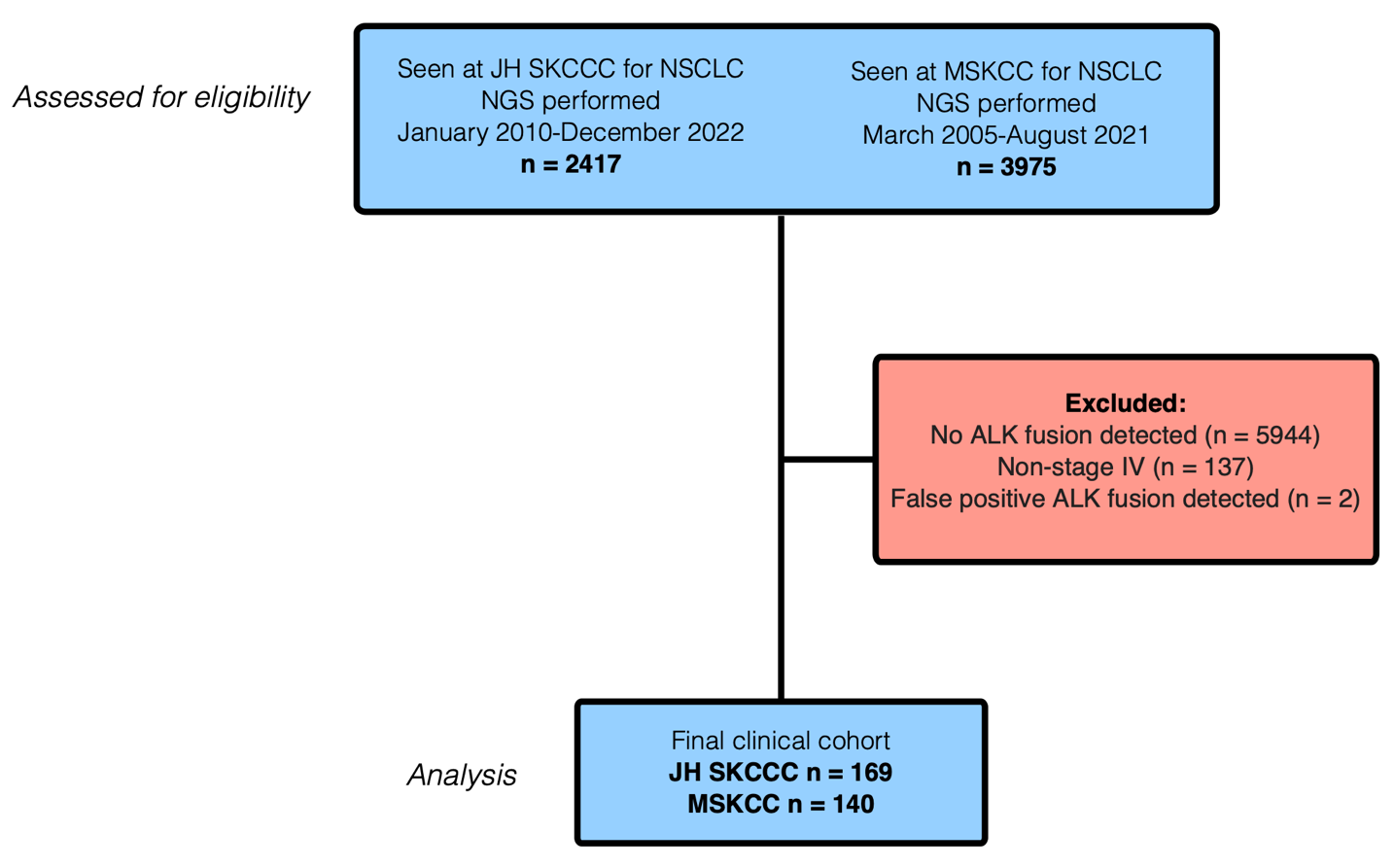


**Supplemental Figure 1:** CONSORT diagram depicting inclusion and exclusion criteria for clinical cohort
