# Supplementary material for: Impact of Tumor-intrinsic Molecular Features on Survival and Acquired Tyrosine Kinase Inhibitor Resistance in ALK-positive NSCLC
Source: Cancer Res Commun. 2024 Mar 14;4(3):786–95. doi: 10.1158/2767-9764.CRC-24-0065 (PMC10939006; doi:10.1158/2767-9764.CRC-24-0065)
Supplement: Supplemental Figure 2 — Distribution of tissue NGS platforms utilized in the clinical cohort. Includes 10 patients who underwent testing with more than one tissue NGS platform. Other commercial NGS platform includes other commercially available platforms. Other institutional NGS platform includes all other academic in house NGS platforms. [file crc-24-0065-s07.docx]

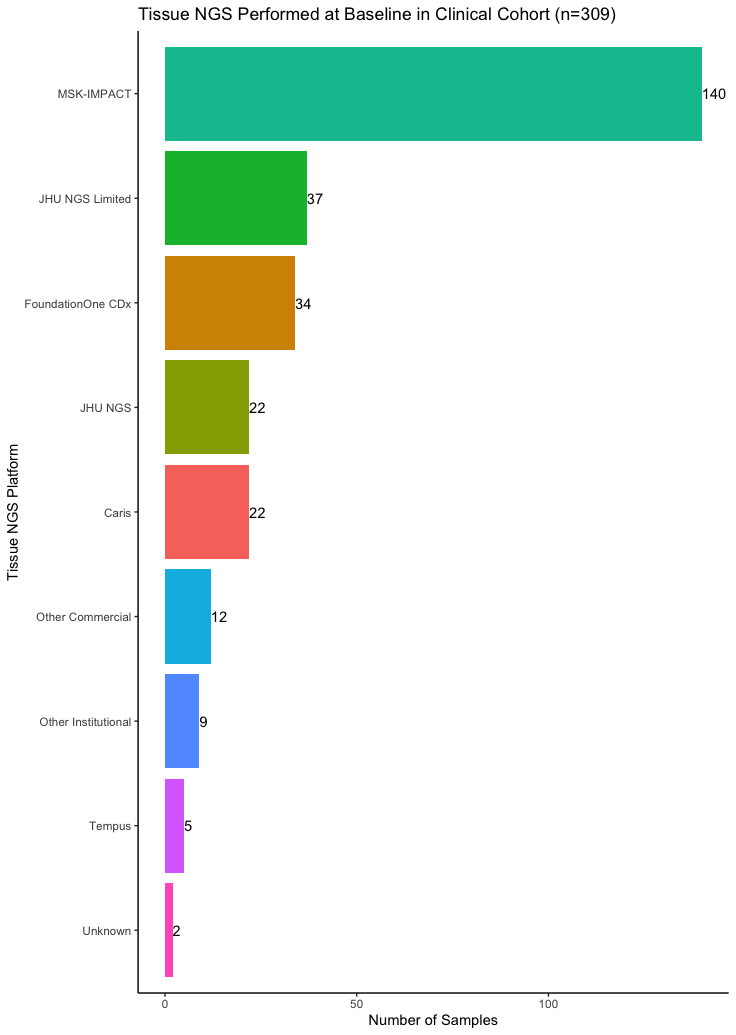


**Supplemental Figure 2:** Distribution of tissue NGS platforms utilized in the clinical cohort. Includes 10 patients who underwent testing with more than one tissue NGS platform. Other commercial NGS platform includes other commercially available platforms. Other institutional NGS platform includes all other academic in house NGS platforms.
