# Supplementary material for: Impact of Tumor-intrinsic Molecular Features on Survival and Acquired Tyrosine Kinase Inhibitor Resistance in ALK-positive NSCLC
Source: Cancer Res Commun. 2024 Mar 14;4(3):786–95. doi: 10.1158/2767-9764.CRC-24-0065 (PMC10939006; doi:10.1158/2767-9764.CRC-24-0065)
Supplement: Supplemental Figure 3 — (A) Samples with single versus multiple ALK resistance mutations in liquid biopsy cohort. (B) Distribution of patients with multiple ALK resistance mutations across the ALK resistance cohort by fusion variant type, with percentages above each variant type column indicate the proportion of patients with multiple ALK resistance mutations. The inset chi-squared statistic demonstrates no significant association between multiple resistance mutations and variant type. (C) Distribution of most common ALK resistance co-mutation pairs [file crc-24-0065-s08.docx]

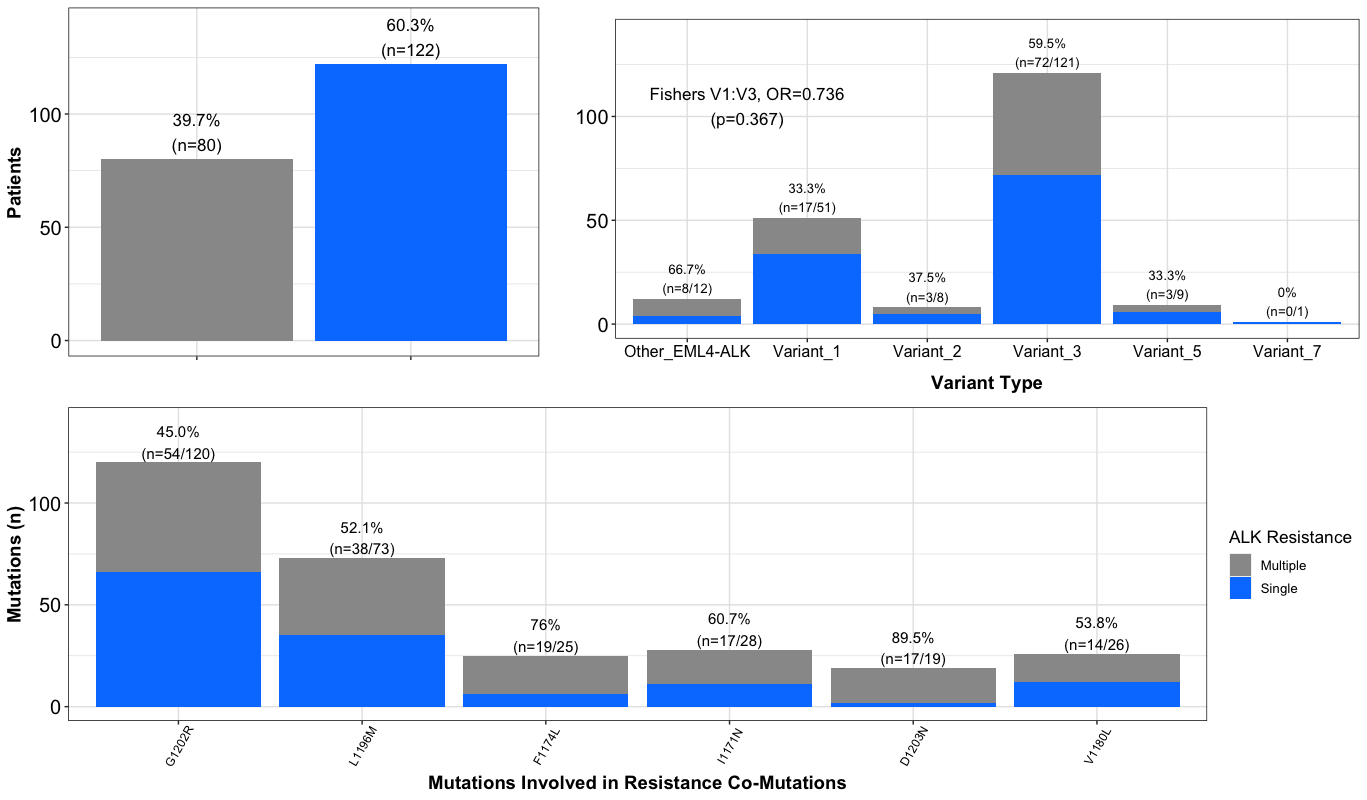


**C**

**B**

**A**

**Supplemental Figure 3:** **(A)** Samples with single versus multiple ALK resistance mutations in liquid biopsy cohort. **(B)** Distribution of patients with multiple ALK resistance mutations across the ALK resistance cohort by fusion variant type, with percentages above each variant type column indicate the proportion of patients with multiple *ALK* resistance mutations. The inset chi-squared statistic demonstrates no significant association between multiple resistance mutations and variant type. **(C)** Distribution of most common ALK resistance co-mutation pairs
