# Supplementary material for: Impact of Tumor-intrinsic Molecular Features on Survival and Acquired Tyrosine Kinase Inhibitor Resistance in ALK-positive NSCLC
Source: Cancer Res Commun. 2024 Mar 14;4(3):786–95. doi: 10.1158/2767-9764.CRC-24-0065 (PMC10939006; doi:10.1158/2767-9764.CRC-24-0065)
Supplement: Supplemental Figure 4 — Clonal and non-clonal ALK resistance mutations across patients with multiple resistance mutations. Each ALK mutation is plotted by sample and colored by putative clonal classification based on the maximum difference between resistance mutation mutant allele frequencies (MAFs). Inset table provides the clonal and non-clonal patient counts for those with v1/v3 variant types. Chi-squared statistic indicates there is no significant association with resistance clonality and fusion type. [file crc-24-0065-s09.docx]

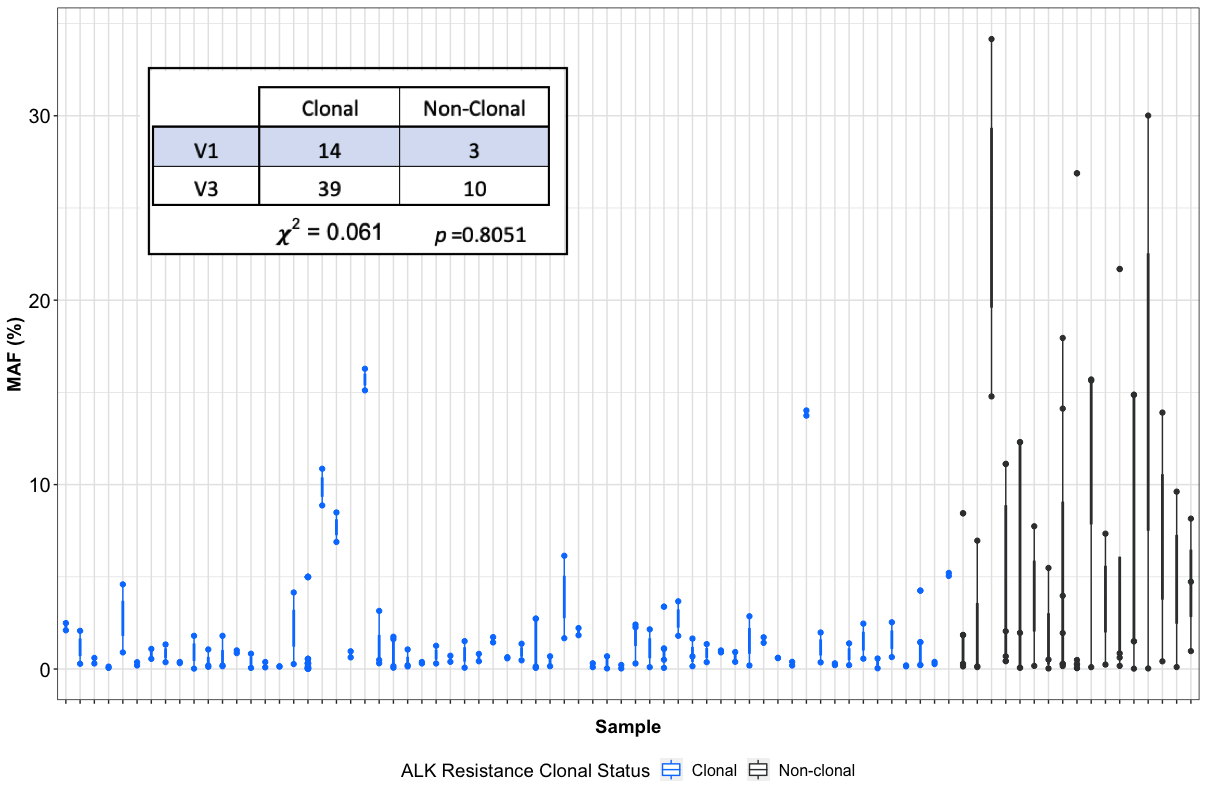


**Supplemental Figure 4:** Clonal and non-clonal ALK resistance mutations across patients with multiple resistance mutations. Each *ALK* mutation is plotted by sample and colored by putative clonal classification based on the maximum difference between resistance mutation mutant allele frequencies (MAFs). Inset table provides the clonal and non-clonal patient counts for those with v1/v3 variant types. Chi-squared statistic indicates there is no significant association with resistance clonality and fusion type.
