# Supplementary material for: Impact of Tumor-intrinsic Molecular Features on Survival and Acquired Tyrosine Kinase Inhibitor Resistance in ALK-positive NSCLC
Source: Cancer Res Commun. 2024 Mar 14;4(3):786–95. doi: 10.1158/2767-9764.CRC-24-0065 (PMC10939006; doi:10.1158/2767-9764.CRC-24-0065)
Supplement: Supplemental Figure 5 — Kaplan-Meier curve for progression-free survival (PFS) on first-line alectinib or brigatinib by (A) variant type, (B) TP53 mutation, and (C) CDKN2A/B mutation status [file crc-24-0065-s10.docx]

**A**
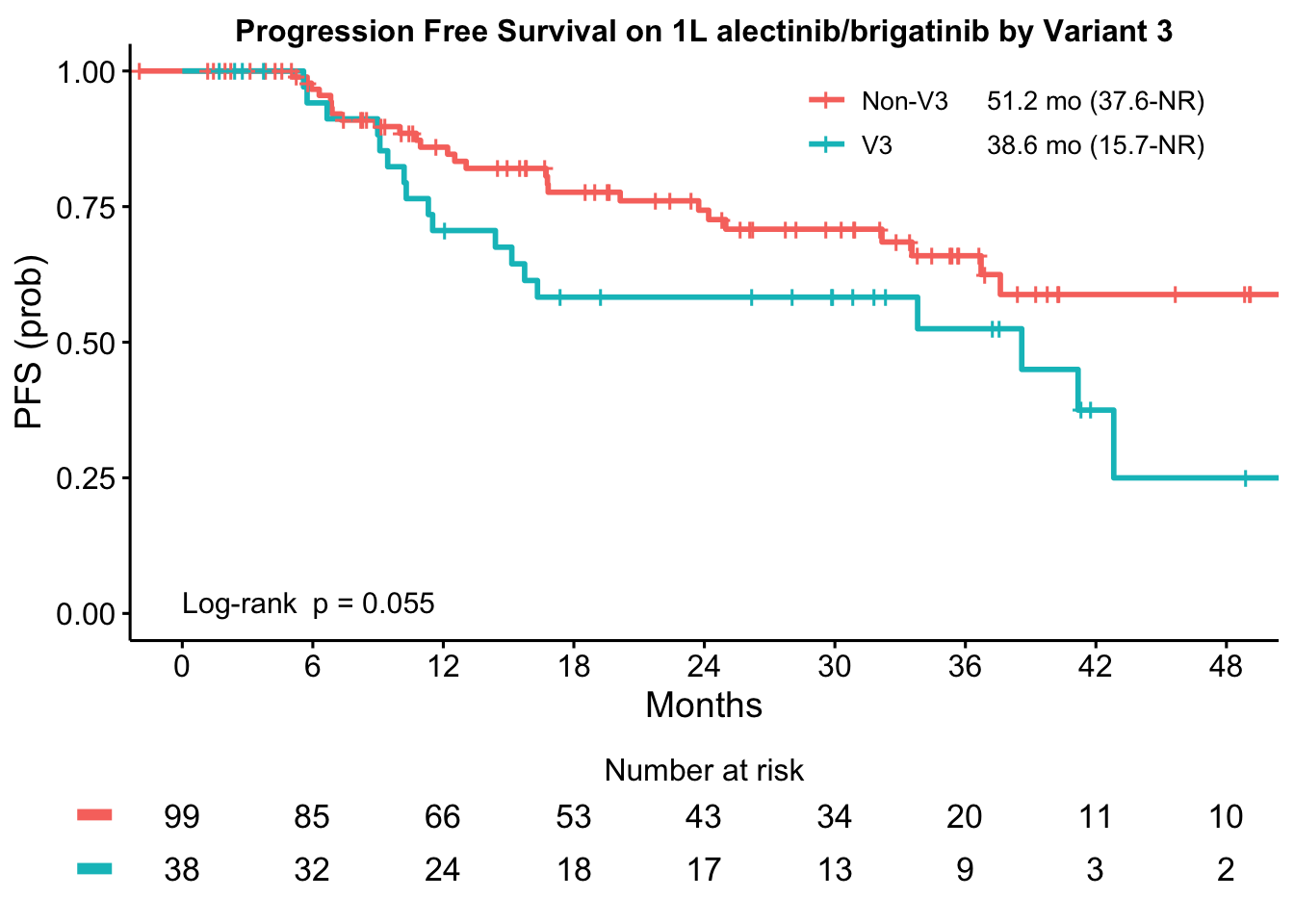


**C**
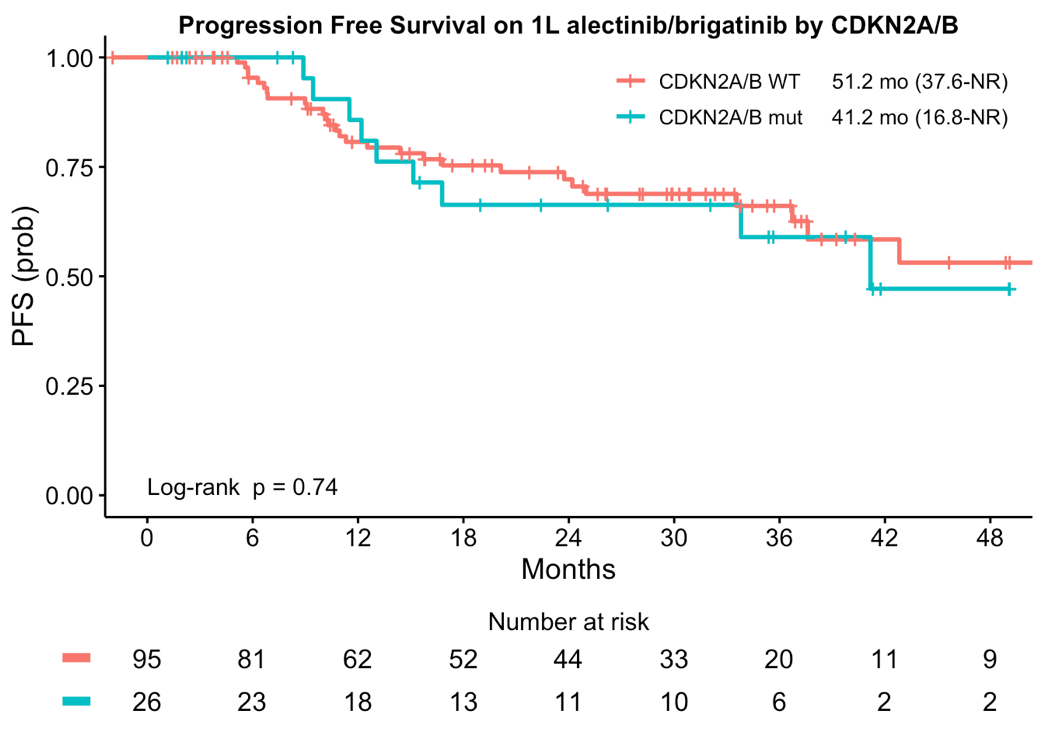


**B**
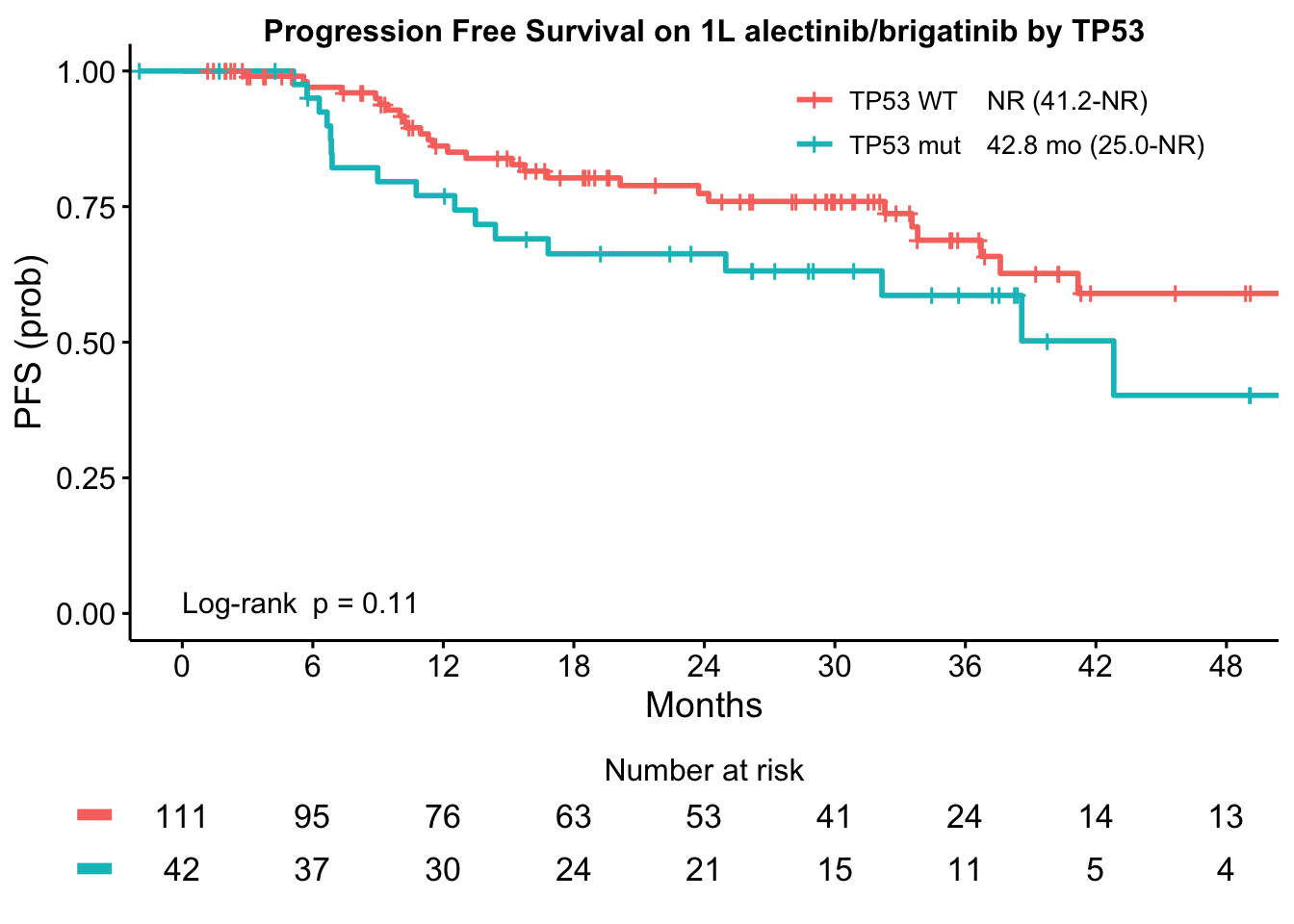


**Supplemental Figure 5:** Kaplan-Meier curve for progression-free survival (PFS) on first-line alectinib or brigatinib by **(A)** variant type, **(B)** *TP53* mutation, and **(C)** *CDKN2A/B* mutation status
