# Supplementary material for: Impact of Tumor-intrinsic Molecular Features on Survival and Acquired Tyrosine Kinase Inhibitor Resistance in ALK-positive NSCLC
Source: Cancer Res Commun. 2024 Mar 14;4(3):786–95. doi: 10.1158/2767-9764.CRC-24-0065 (PMC10939006; doi:10.1158/2767-9764.CRC-24-0065)
Supplement: Supplemental Figure 6 — Kaplan-Meier curve for (A) Overall survival (OS), (B) first-line TKI progression-free survival (PFS) and (C) PFS on first-line alectinib or brigatinib by PD-L1 high (TPS > 50%) status [file crc-24-0065-s11.docx]

**A**
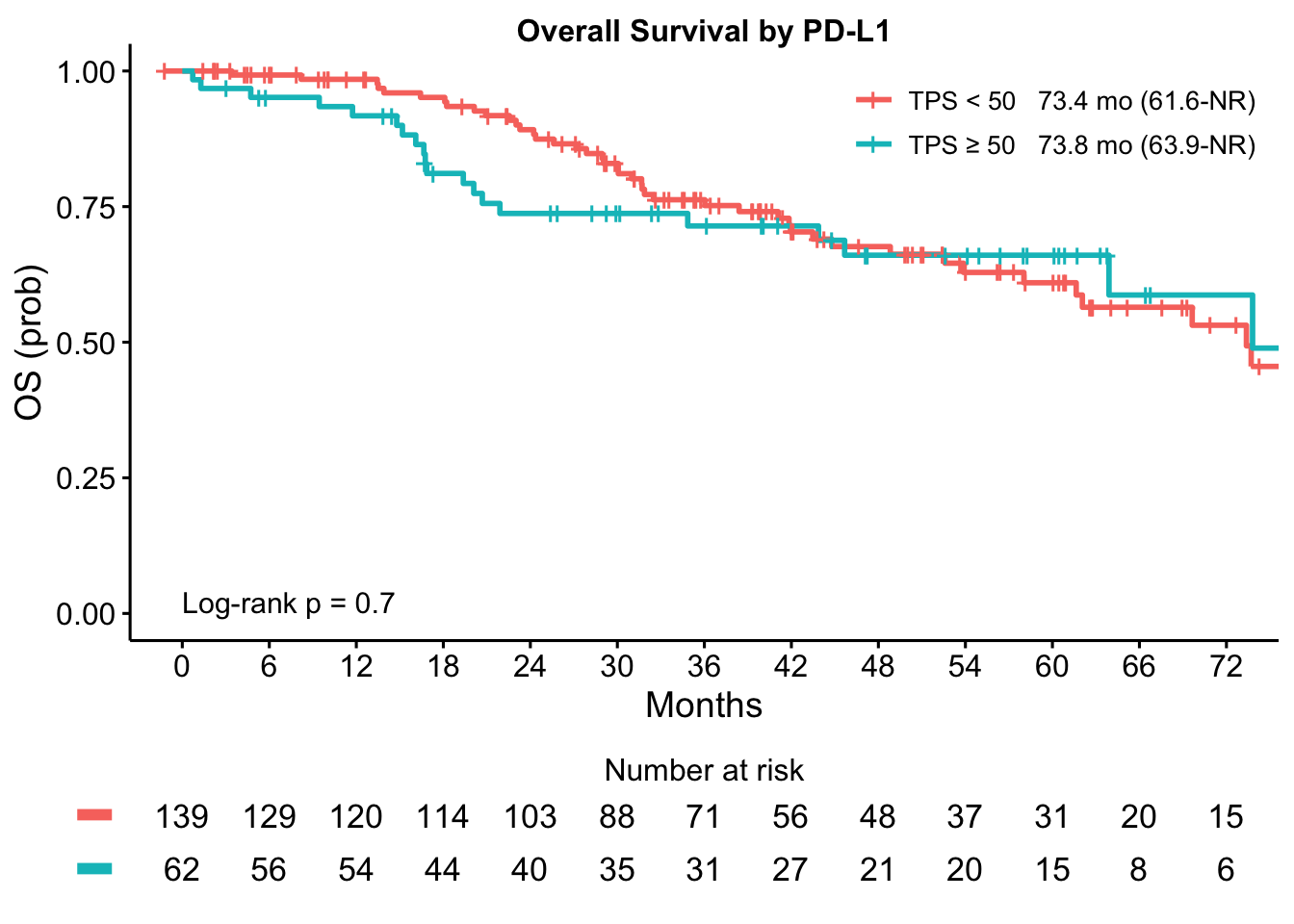


**C**
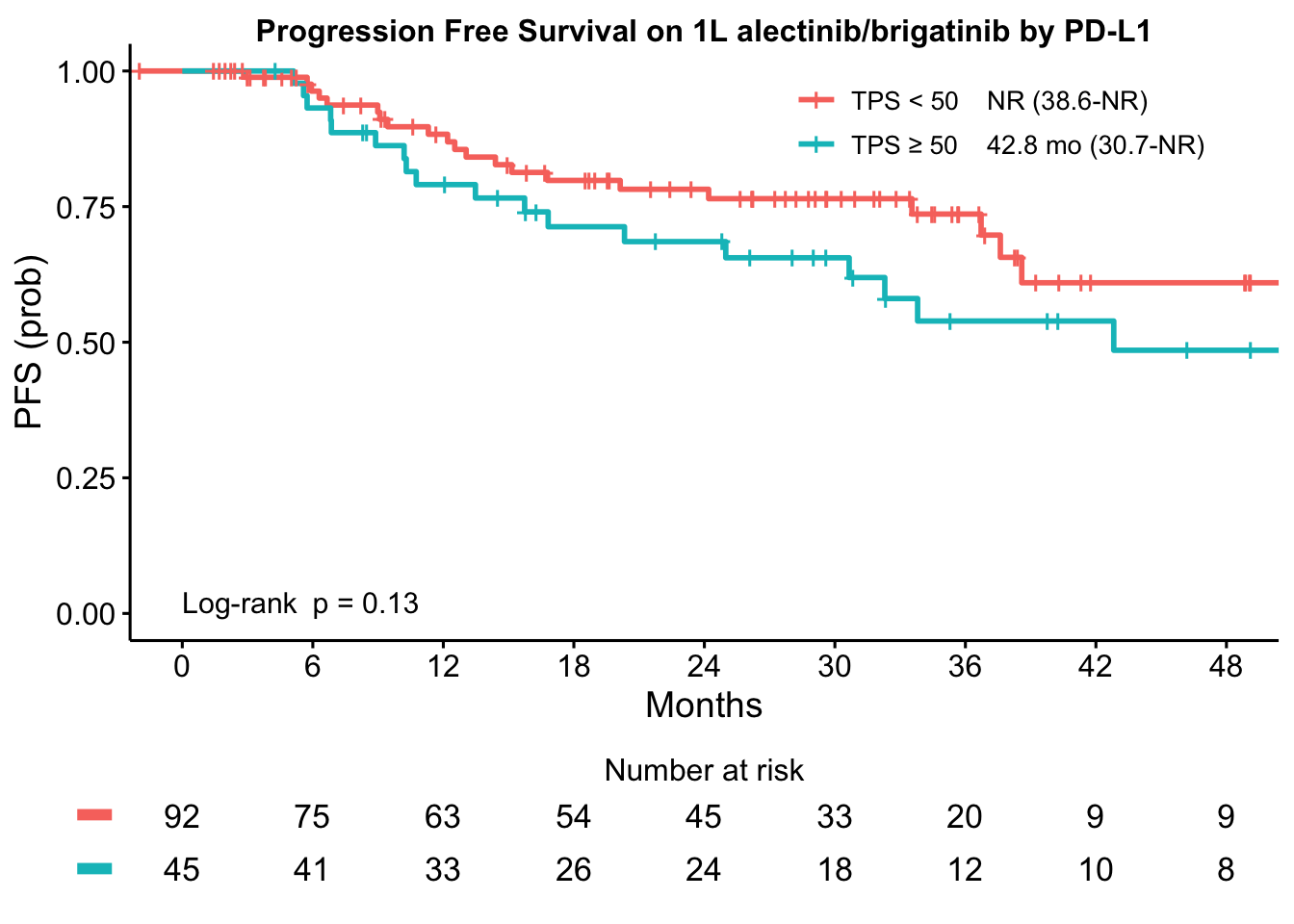


**B**
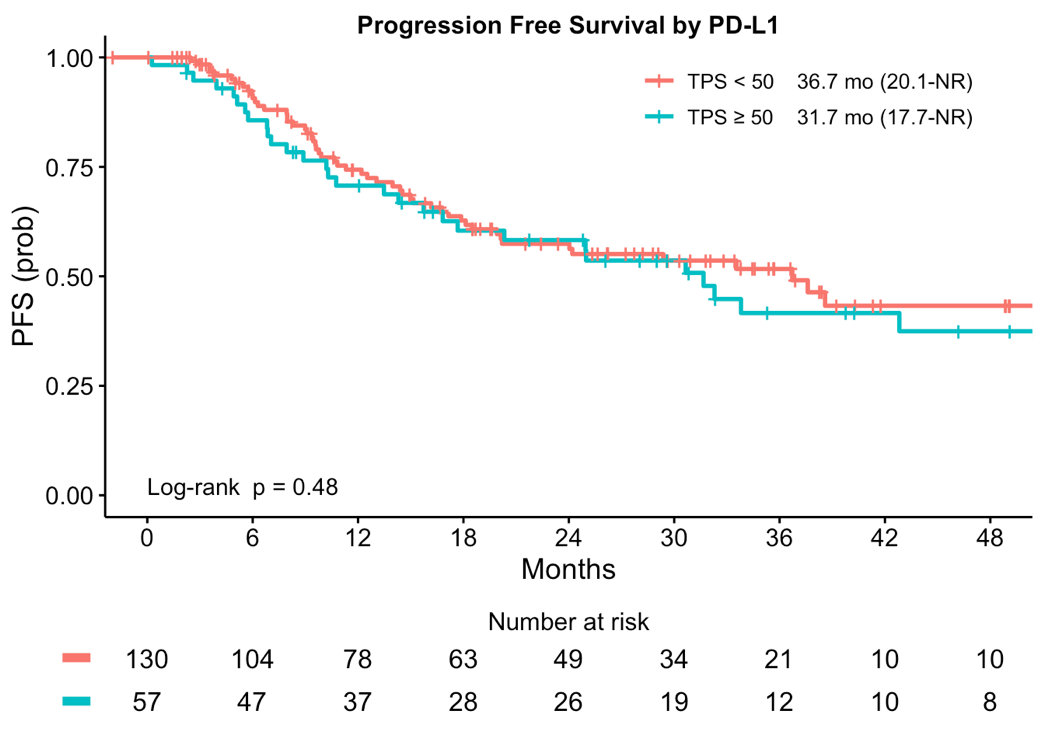


**Supplemental Figure 6:** Kaplan-Meier curve for **(A)** Overall survival (OS), **(B)** first-line TKI progression-free survival (PFS) and **(C)** PFS on first-line alectinib or brigatinib by PD-L1 high (TPS > 50%) status
